# Non-Aerated Common Nettle (*Urtica dioica* L.) Extract Enhances Green Beans (*Phaseolus vulgaris* L.) Growth and Soil Enzyme Activity

**DOI:** 10.3390/life12122145

**Published:** 2022-12-19

**Authors:** Branka Maričić, Mia Brkljača, Dean Ban, Igor Palčić, Kristijan Franin, Šime Marcelić, Smiljana Goreta Ban

**Affiliations:** 1 Department of Ecology, Agronomy and Aquaculture, University of Zadar, Trg kneza Višeslava 9, 23000 Zadar, Croatia; 2 Mia Brkljača, 23000 Zadar, Croatia; 3 Institute of Agriculture and Tourism, Karla Huguesa 8, 52440 Poreč, Croatia

**Keywords:** acid phosphatase, alkaline phosphatase, dehydrogenase, legumes, nodulation, soil fertility

## Abstract

One of the limiting factors in organic farming is the scarcity of allowed fertilizers and chemicals for plant protection. Plant and compost extracts are a promising solution for fertilization because of their positive effect on plant growth and soil microbial activity. Nettle extract was already successfully applied to some vegetables. Not-aerated nettle extract, obtained from dry nettle leaves, was applied in experiments with green beans in a quantity of 1 L per pot at two-day intervals was studied. A three-factorial experimental design was applied with two soil types (brown—Calcic Gleysol and red—Eutric Cambisol), soil disinfection with dazomet or not, and irrigated with nettle extract or water. Nettle extract application increased all above-ground traits; plant height, leaf area, flower buds, shoot dry weight at flowering, pod length, pod diameter, and shoot dry weight at harvest by 49%, 66%, 43%, 36%, 11%, 9%, and 37%, respectively, the root length at harvest by 59%, total yield by 48%, soil respiration by 91% and 74% in two soil types, and alkaline phosphatase by 30%. Dehydrogenase activity was enhanced by nettle extract application on red soil, while nettle extract application had no effect on root nodulation. The nettle extract application benefits in green bean organic production were attributed to the nutrients and other components present in the extract and not to nitrogen fixation. The optimization of the dose of the extract and experiments in real conditions of green bean production would be the next step toward the implementation of nettle extract as an organic fertilizer.

## 1. Introduction

Conventional agriculture has an important role in improving crop productivity to meet food demands by humans and domestic animals. In order to keep high productivity, it largely relays on the use of chemical products for plant nutrition and protection. The research towards finding quality ways to fertilize plants by organic methods is important to gradually move from an agricultural management practice that causes problems such as soil depletion, erosion, biodiversity loss, and crop quality to management that will be more sustainable and environmentally friendly with sufficient yield [1,2]. Nevertheless, the limiting factor in organic farming is the scarcity of the allowed fertilizers or chemicals for plant protection.

Soil health, defined as the continued capacity of the soil to function as the living ecosystem that sustains diverse organisms [3], has indispensable importance in managing soil productivity. Soil type affects the plant’s water and mineral uptake and root-zone temperature and thus influences plant growth and physiology [4]. To preserve soil health, treatments such as soil disinfection should be applied sometimes. Research reported that even in healthy soil, plants showed increased growth after soil disinfection was applied [5]. Soil organic matter affects the growth and yield of plants by supplying them with nutrients directly or indirectly and by changing the physicochemical properties of the soil. Solid organic fertilizers such as manure and straw are conventionally used to increase soil organic matter.

Interest in liquid organic fertilizers research is still unabated. Those are extracts of different plant materials or extracts of composts obtained after aerated or not aerated extraction [6,7,8]. Such fertilizers primarily increase the abundance of soil microorganisms’ populations [6] and their biodiversity [9] and improve conditions in the rhizosphere that encourage plant growth [6,10,11,12]. In addition, liquid organic fertilizers contain easily available nutrients and water, while biopolymers such as humic and fulvic acids. Liquid organic extracts made from compost positively affect the yield and quality of cultivated plants [8,13]. Co-application of aerated and non-aerated compost extract with organic and inorganic fertilizers enhanced yield compared with the sole application of inorganic fertilizer [8]. Liquid organic fertilizers induce higher root growth, modified root architecture, and microbial populations in the rhizosphere of cultivated plants in comparison to unfertilized soil or the application of mineral fertilizer [9,14].

Plant extracts, used as liquid organic fertilizers and containing substances that promote natural processes that enhance plant growth, are called biostimulants [2]. Biostimulants are substances that improve nutrient uptake, improve plant resistance to stress, positively affect their primary and secondary metabolism, and modify physiological processes, as well as their yield [15]. Herbal biostimulants include extracts of seaweed and plant species [16,17] and usually are applied as a liquid in the form of natural extracts [2]. The various plant extracts stimulated the growth of cabbage seedlings [18], and vegetable extracts rich in phenols and hormones increased the root and leaf biomass, chlorophyll, and sugar content of maize seedlings [19]. Biostimulants from moringa extracts added to mineral fertilizers enhanced the growth and quality of pea plants [20].

Nettle (*Urtica dioica* L.) is a plant that is often used in medicine [21,22,23,24], in the cosmetics industry [25], and in the textiles industry for the purpose of obtaining solid fibers [26,27]. Due to its rich chemical composition and high nutritional value, nettle is used for human and also animal nutrition [27].

Many benefits of nettle have been found in agricultural use. Nettle extract was effective as a biostimulant for cabbage seedlings growth [2] and the growth of lettuce radicle [28], probably due to its auxin content [6]. Fertilization poured into the soil with nettle extract was equivalent to foliar fertilization regarding the yield and vegetative parameters (plant height, diameter stem) of green beans [29]. However, nettle extract application suppresses the spread of plant pathogens also [30,31,32]. It is rich in boron [33], sulphur, calcium, phosphorus, nitrogen [34], phenolic compounds, antioxidants [35], and chlorophyll [36]. Additionally, nettle extract stimulates the uptake of nitrogen but has no effect on the uptake of phosphorus and potassium [37].

Green beans (*Phaseolus vulgaris* L.) are a food rich in polyphenols and, as such, have a positive effect on human health in controlling obesity, diabetes, and inflammatory processes in the body [38]. Also, it contains a great amount of vitamin C, dietary fiber, carbohydrates, minerals, and proteins [39]. Green beans have a very short vegetation time (60 to 80 days) and are easily grown on different soil types. It can be grown at higher and lower altitudes and in dry and humid climates [40].

Since nettle is a widespread and easily accessible plant, its application in organic agriculture can be a feasible solution for the enrichment of soil with plant nutrients and enhancement of soil microbial activity. However, according to accessible literature, the effect of its application on plant growth and yield is inconsistent, and there is a general lack of information on its effect on soil properties. Therefore, the aim of this study was to determine how nettle extract affects soil fertility, nodulation, vegetative growth, and yield of green beans.

## 2. Materials and Methods

### 2.1. Experimental Site and Experiment Design

A three-factorial experimental design was set up with the following factors: (a) soil type (brown or red); (b) soil disinfection (yes or no); and (c) soil irrigation (water or nettle extract).

The brown soil and red soil were chosen for the experiment due to their different pedogenesis. Brown soil was classified as hydro-meliorated Calcic Gleysol, and red soil was classified as Eutric Cambisol (trivial name Terra Rossa) [41]. Both soils were previously used for vegetable production. The chemical composition of these two soil types at the beginning of the experiment was reported in Table 1. The red soil had a slightly higher pH but was better supplied with P and K compared to the brown soil.

For soil disinfection treatment, the pesticide dazomet or 3,5-Dimethyl-1,3,5-thiadiazinane-2-thione (Basamid, Kanesho Soil Treatment, Belgium) was applied at 250 g/m^3^ according to manufacturer instructions against weeds, pathogen fungi, and nematodes.

Nettle was collected from a natural habitat around Pula, Croatia (N 44.905, E 13.957). Nettle leaves were cut during midday and dried in a forced air dryer at 30 °C for at least 24 h. The extract was prepared weekly by soaking 183 g of dry nettle in 10 L of tap water in closed PE containers for 14 days with occasional mixing and kept at ambient temperature [4]. The chemical composition of nettle extract is reported in Table 2. Undiluted nettle extract had greater amounts of NH_4_-N, K, Ca, B, Mn, Zn, Cu, and Mo, similar amounts of P, Mg, S, and Fe, and lower amounts of NO_3_-N compared to Hoagland’s nutrient solution [42] (Table 2). The nettle extract prepared for this experiment was supplied with NH_4_-N, P, S, Fe, Mn, Zn, Cu, and Mo below the lower limits reported by Peterson and Jensen [6]. However, K, Ca, and Mg were in the reported range.

The prepared nettle extract was strained and diluted 1:3 with tap water. It was applied in a dose of 1 L of diluted nettle extract per pot at two-day intervals. The extract, after dilution, had a pH value of 6.5–7.0. Chemicals or other amendments except nettle and water were not applied during the experiment.

Green beans var. ‘Top crop’ were grown in 3-L PE containers filled with brown or red soils in a non-heated greenhouse in Zadar, Croatia (N 44.077, E 15.288).

Six green bean seeds were sown on 3 May and irrigated with tap water until the emergence of the first true leaf (31 May). Thereafter, the plants were irrigated with tap water or nettle water extract for four weeks according to plant demands.

### 2.2. Plant Sampling

Plant samples were collected at the flowering stage 43 days after sowing. Stems were cut 1 cm above the highest visible roots. The plant height was measured as the distance from the first node to the apical bud on the main stem. The total number of flower buds per plant was counted.

Above-ground plant mass was washed in deionized water and blotted dry using a paper towel. All leaves larger than 1 cm were scanned, and leaf area was measured using ImageJ software (ImageJ, US National Institutes of Health, Bethesda, MD, USA). Leaves and stems for dry weight determination were dried in a drying oven at 70 °C for 48 h.

Roots were carefully washed with tap water over the sieve having 0.2 mm openings, and the root nodules were immediately counted. Roots free of soil particles were spread out in a transparent tray containing a thin layer of water and scanned. Roots were scanned with a digital image analysis system at a resolution of 300 dots per inch (dpi), and total root length and distribution by diameter (0–0.25; 0.25–0.50; 0.50–0.75; 0.75–1.0; 1.0–1.25 and >1.25 mm) were measured (WinRhizo LA 1600, Régent Instruments Inc., Quebec City, QC, Canada). Scanned roots were blotted and dried in an oven as was described for stem and leaves. Specific root length (root length/root dry weight) was calculated for all samples.

The vegetative traits of green bean plants were again measured at the end of the experiment, together with yield, pod length, and diameter.

### 2.3. Soil Chemical Analysis

Soil chemical analysis was performed at the flowering stage. From each pot, roots were removed, and soil was thoroughly homogenized and air-dried. The soil was crushed and passed through a 2-mm sieve. The soil was analyzed for active reaction (pH of soil:water suspension of 1:2.5) and potential soil reaction (pH of soil: 1 M KCl suspension of 1:2.5) [43], soil organic matter by the method of Walkley-Black [44], soil-available P and K by ammonium lactate method [45], soil conductivity in soil:water suspension of 1:5 [46]. Total carbonate was determined using the volumetric calcimeter method, and active carbonate was determined by the reaction of carbonate with 0.1 M oxalate following the acidification with H_2_SO_4_ and reaction with KMnO_4_ [47].

#### Soil Enzymatic Activity and Soil Respiration

At full bloom of the green bean plants, soil samples for enzymatic activity and soil respiration were taken. The soil was randomly sampled across the container, placed in PE bags, and kept at 4 °C until further analysis.

Acid and alkaline phosphatase activities were determined according to Tabatabai [48]. One gram of wet soil was incubated with 0.2 mL of toluene, 1 mL of 0.05 M p-nitrophenyl phosphate, and 4 mL of MUB buffer at pH 6.5 for acid phosphatase or at pH 11.0 for alkaline phosphatase at 37 °C for 1 h. Incubation was stopped with the addition of 1 mL of 0.5 M CaCl_2_ and 4 mL of 0.5 M NaOH and filtered. The p-nitrophenol was measured spectrophotometrically at 410 nm. Data were expressed on an oven-dry soil basis (105 °C).

Dehydrogenase activity was determined according to the modified method reported by Wolinska, Stępniewska, and Szymańska [49]. One gram of wet soil was incubated with 20 mg of CaCO_3_, 0.5 mL of 1% glucose, and 0.2 mL of 3% triphenyl tetrazolium chloride (TTC) at 30 °C for 20 h. The triphenyl formazan (TPF) formed was extracted by adding 5 mL of ethanol and shaking vigorously for 30 s. The suspension was filtered and diluted to 10 mL with ethanol. TPF was measured spectrophotometrically at 485 nm. Data were expressed on an oven-dried soil basis (105 °C).

Soil respiration measurement was conducted according to the protocol established by Rubio [50]. Air-dried soil (32 g) was placed in a 1-L glass jar and brought to a water field capacity of 45%. The glass beaker containing 20 mL of 0.2 M NaOH was placed inside the jar. The jar was immediately tied and incubated for 7 days at 22 °C in a growth chamber. The soil respiration was measured by titrating NaOH solution with 0.1 N HCl after adding 2 mL of 0.5 M BaCl_2_ and 0.1% phenolphthalein.

### 2.4. Statistical Analysis

Plant traits were measured at the flowering stage, while yield, pod length, pod diameter, and shoot and root dry weight were measured at the end of the experiment. The effects of soil type, disinfection, and irrigation were analyzed as between-subject independent variables on leaf area, shoot dry weight, plant height, and enzyme activity as continuous dependent variables using the aligned rank transform for nonparametric factorial analyses using ARTool (ARTool, University of Washington, Washington, DC, USA). After the alignment and ranking, the means of dependent variable groups were analyzed by three-way ANOVA. The association between independent variables was determined by a nonparametric Spearman rank-order correlation test.

## 3. Results

### 3.1. Soil Physicochemical Properties

**Figure 1:**
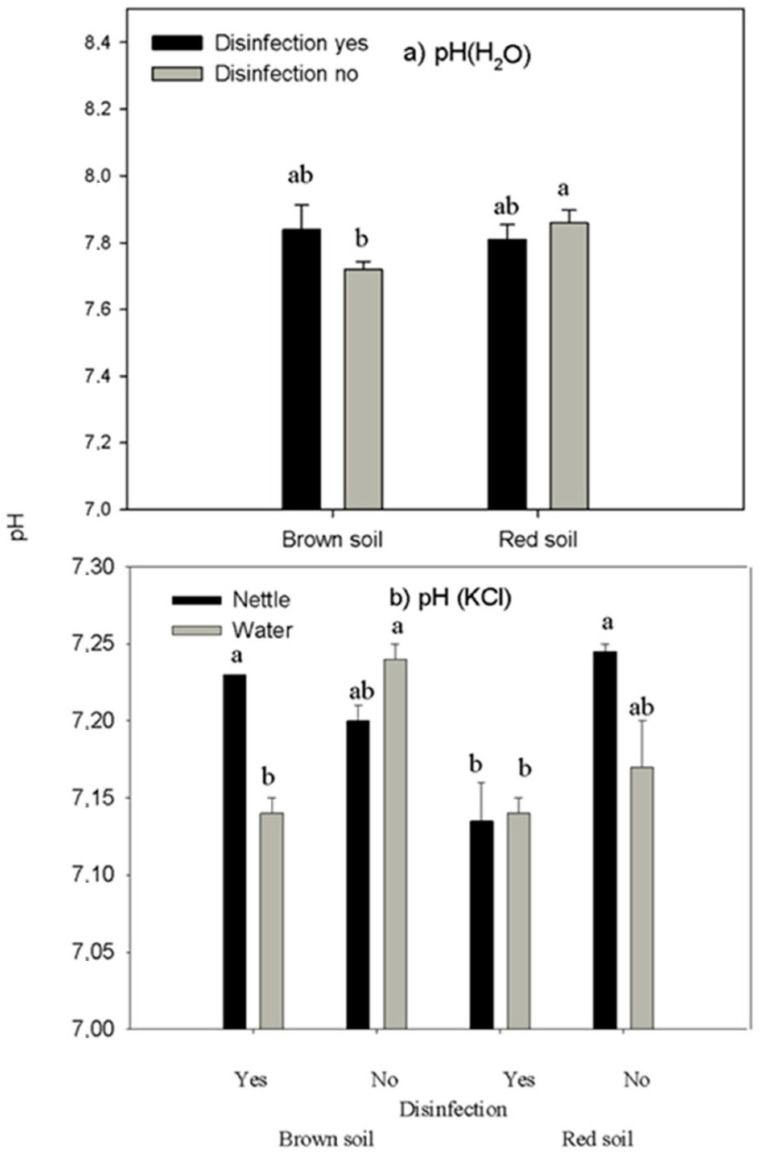
The effect of the interaction between soil type and disinfection on (**a**) pH H_2_O and the interaction of soil type, disinfection, and irrigation on (**b**) pH KCl. Different letters mark statistically different treatments at a 0.05 level of significance.

Soil organic carbon content was not affected by soil type, disinfection, or irrigation treatment. Soil active acidity, as indicated by soil solution (pH H_2_O), was affected by irrigation (Table 3, *p* = 0.00114) and was lower with water irrigation than with nettle irrigation. It was also affected by the interaction between soil type and disinfection (Table 3, *p* = 0.0022) and was significantly lower in no-disinfected brown soil than in no-disinfected red soil, while other interactions with soil were similar in both soil types (Figure 1a).

The potential acidity of soil or soil buffer capacity, as indicated by acidity due to hydrogen ions from the clay minerals (pH KCl), was affected by the interaction between irrigation, disinfection, and soil type (Table 3, *p* = 0.001104), and was lower in both irrigations of disinfected red soil, and water-irrigated disinfected brown soil than in nettle-irrigated no-disinfected red soil, water-irrigated no-disinfected brown soil, and nettle-irrigated disinfected brown soil (Figure 1b).

### 3.2. Soil Nutrients

**Figure 2:**
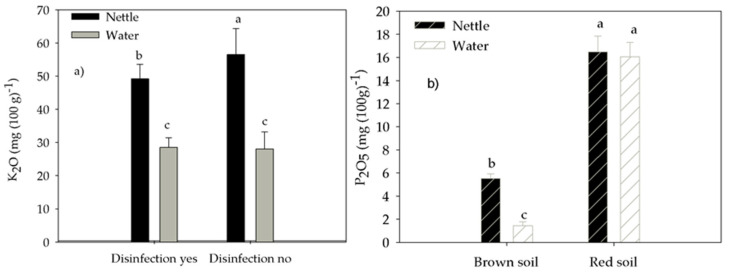
The effect of the interaction between disinfection and irrigation on (**a**) soil-available K and the interaction of soil type and irrigation on (**b**) soil-available P. Different letters mark statistically different treatments at a 0.05 level of significance.

**Figure 3:**
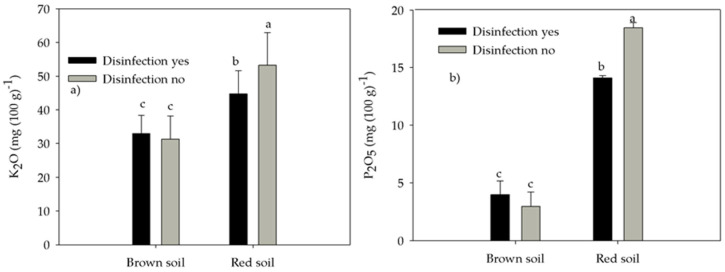
The effect of the interaction between soil type and disinfection on (**a**) soil-available K and (**b**) P. Different letters mark statistically different treatments at a 0.05 level of significance.

Soil-available K content was affected by interactions between irrigation and disinfection (Table 3) and was greater in nettle-irrigated no-disinfected soil than in nettle-irrigated disinfected soil, water-irrigated soil disinfected or no-disinfected soil by 15%, 98%, and 101%, respectively (Figure 2a). Soil-available K was also affected by the interaction of soil type and disinfection (Table 3) and was greater in red no-disinfected soil than in red disinfected soil, brown disinfected, and no-disinfected soil by 19%, 61%, and 60%, respectively, and two brown soils were similar (Figure 3a).

Soil-available P was affected by the interaction between soil type and irrigation (Table 3), and in nettle-irrigated red soil was similar to water-irrigated red soil but was greater in nettle-irrigated brown soil and water-irrigated brown soil by 49% and 26%, respectively (Figure 2b). Soil-available P was also affected by the interaction between soil type and disinfection (Table 3), and, similarly to soil-available K, was greater in red no-disinfected soil than in red disinfected soil, brown disinfected and no-disinfected soil by 31%, 3.6 times and 5.2 times, respectively, and two brown soils were similar (Figure 3b).

### 3.3. Plant Functional Traits

In samples collected at the flowering stage, nettle extract application increased shoot biomass weight and root:shoot ratio compared with water irrigation by 36% and 45%, respectively (Table 4).

**Figure 4:**
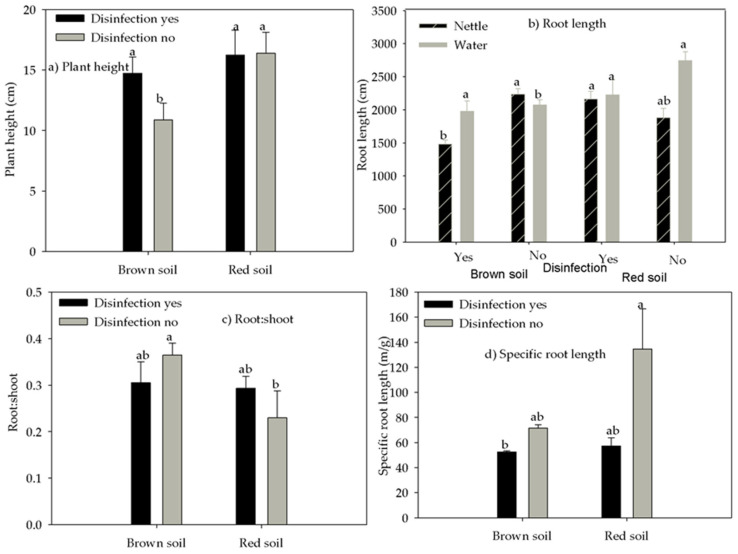
The effect of the interaction between soil type and disinfection on (**a**) plant height, (**c**) root:shoot, and (**d**) specific root length, and the effect of the interaction between irrigation, soil type, and disinfection on (**b**) root length. Different letters mark statistically different treatments at a 0.05 level of significance.

Root:shoot ratio was affected by the interaction between soil type and disinfection (Table 4). The ratio of root:shoot was higher in brown no-disinfected soil than in red no-disinfected soil by 50% (Figure 4c). The specific root length (SRL—the ratio of root length to dry root mass) was significantly smaller in brown disinfected soil than in red no-disinfected soil by 61% (Figure 4d).

Plant height was smaller in no-disinfected brown soil by 33% compared with the same soil disinfected, while plant height was similar in red soil regardless of disinfection (Figure 4a).

Among below-ground functional traits, significant interactions between irrigation, disinfection, and soil type were observed for root nodule count and root length (*p* < 0.05). Root nodule count (Table 5) was significantly higher in water-irrigated disinfected brown soil than in all other treatments, which had a similar nodule count and was 4.7 times greater compared with the same nettle-irrigated soil.

Root length was greater in water-irrigated, no-disinfected red soil, nettle-irrigated disinfected red soil, water-irrigated disinfected brown soil, and nettle-irrigated no-disinfected brown soil compared with nettle-irrigated disinfected brown soil and water-irrigated no-disinfected brown soil. The enhancement was, compared with nettle-irrigated brown disinfected soil, 85%, 34%, 46%, and 51%, respectively (Figure 4b).

The application of soil disinfection increased the leaf area by 50% and the flower bud count by 25% compared to no-disinfected soil (Table 5). 

Root length per every 0.5 mm of root diameter revealed that the thinnest roots (0.00–0.50 mm root diameter) were longer in no-disinfected soil, while the roots of thicker diameters (>4.5 mm, 4.0–4.5 mm, 3.0–3.5 mm, 2.5–3.0 mm and 2.0–2.5 mm) were longer in disinfected soils (Figure 4d).

Among vegetative traits, only flower bud count was affected by soil type (*p* = 0.0063) and was greater in red than in brown soil by 19% (Table 5).

### 3.4. Plant Growth and Production

The effects of soil type, disinfection, and irrigation on pod length and pod diameter, total yield, and dry weight of roots and shoots at the end of the experiment were presented in Table 6.

Irrigation affected pod length and diameter, as well as total yield (*p* < 0.05), and was greater with nettle irrigation than with water irrigation by 11%, 9%, and 48%, respectively (Table 7).

Shoot dry weight was affected by the interaction between disinfection and irrigation (*p* = 0.0119) and was smaller in no-disinfected water-irrigated soil than in nettle and water-irrigated disinfected soil, and in nettle-irrigated no-disinfected soil by 44%, 35%, and 42%, respectively (Table 7). 

Root dry weight was affected by the interaction of soil and irrigation (*p* = 0.0039) and was greater in nettle-irrigated red soil than in water-irrigated red soil and nettle and water irrigations in brown soil by 34%, 81%, and 72%, respectively (Table 7).

Total yield was also affected by soil type (*p* = 0.014) and was greater in red soil than in brown soil by 49%. It was also affected by disinfection (*p* < 0.01) and was greater in disinfected soil than in no-disinfected soil by 2.2 times. Disinfection affected pod length (*p* < 0.01), which was greater in disinfected soil by 11%. Pod diameter was affected by the interaction of soil type and disinfection (*p* < 0.001) and was smaller in no-disinfected brown soil than in disinfected or no-disinfected red soil and disinfected brown soil by 14%, 18%, and 14%, respectively (Table 7).

### 3.5. Soil Enzymatic Activity

The effects of soil type, disinfection, and irrigation on the activity of dehydrogenase (DHA), acid (AcP), and alkaline phosphatase (AlP) were presented in Table 8. 

**Figure 5:**
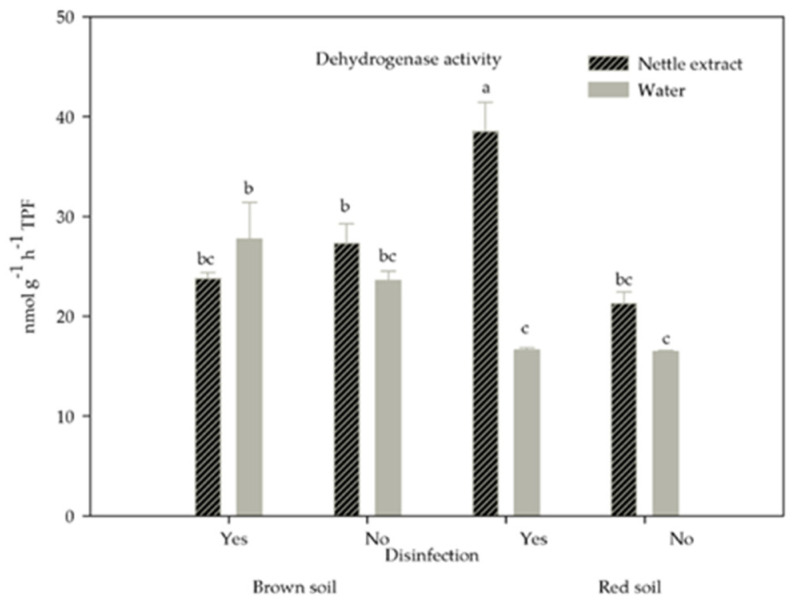
The effect of the interaction between soil type, disinfection, and irrigation on dehydrogenase activity. Different letters mark statistically different treatments at a 0.05 level of significance.

The activity of dehydrogenase was affected by the interaction between irrigation, soil type, and disinfection (*p* = 0.0011). The DHA was enhanced in nettle-irrigated disinfected red soil compared with water-irrigated disinfected red soil and water-irrigated no-disinfected red soil, 2.3 times and 1.2 times, respectively, while DHA was similar in brown soil regardless of disinfection and irrigation (Figure 5).

The alkaline phosphatase was affected by irrigation (*p* = 0.0011) and was higher with nettle irrigation than with water irrigation by 30%. The activities of acid and alkaline phosphatase were affected by soil type (*p* < 0.05), and both were greater in brown soil (Table 8), compared with red soil by 24% and 58%, respectively.

### 3.6. Soil Respiration

**Figure 6:**
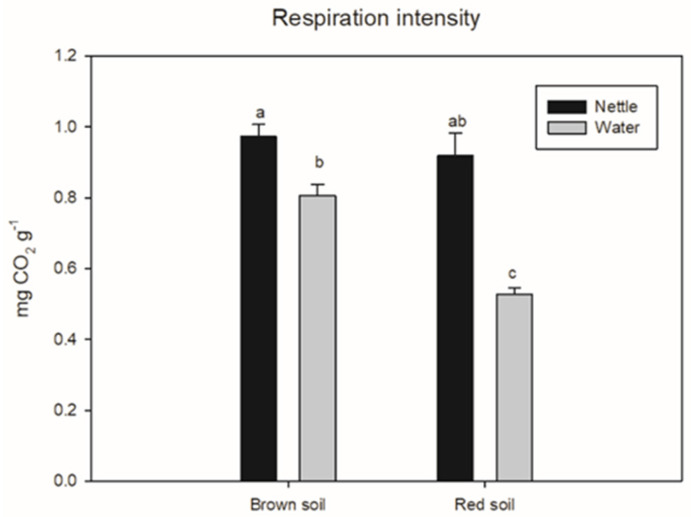
The effect of the interaction between soil type and irrigation type on soil respiration intensity after seven days of incubation at 22 °C. Different letters mark statistically different treatments at a 0.05 level of significance.

Soil respiration was affected by the interaction between irrigation and soil type (*p* = 0.0284). It was greater in nettle-irrigated brown soil than in water-irrigated red and brown soil, and the enhancement was 91% and 25%, respectively (Figure 6).

### 3.7. Correlation Analyses

The correlation analysis of plant growth and soil parameters measured at the flowering stage was given in Table 9. Green bean growth parameters were significantly related to some soil parameters. Plant height was positively related to soil parameters of available P and K (Pearson coefficient = 0.58 and 0.84, respectively). Leaf area was positively related to dehydrogenase activity and soil-available K (Pearson coefficient = 0.54 and 0.57, respectively). Flower number was positively related to soil-available K (Pearson coefficient = 0.66). Root nodule count was negatively related to electrical conductivity, organic matter, and potential acidity (pH KCl) (Pearson coefficient = −0.58, −0.57, and −0.51, respectively) and was positively related to pH H_2_O (Pearson coefficient = 0.79). Root:shoot ratio was negatively related to soil-available P and K (Pearson coefficient = −0.58 and −0.57, respectively). Root:shoot ratio was negatively related to specific root length (Pearson coefficient = −0.66).

Among soil parameters, we observed some significant relations. Electrical conductivity (EC) was positively related to pH KCl, soil respiration, and alkaline phosphatase (Pearson coefficient = 0.56, 0.76, and 0.75, respectively) and negatively related to pH H_2_O (Pearson coefficient = −0.85). pH H_2_O was negatively related to organic carbon, soil respiration, and alkaline phosphatase activity (Pearson coefficient = −0.60, −0.56, and −0.58, respectively). Soil-available P was positively related to soil-available K (Pearson coefficient = 0.68) and negatively related to activities of acid and alkaline phosphatase (Pearson coefficient = −0.51 and −0.74, respectively). Soil respiration was positively related to the activities of dehydrogenase and alkaline phosphatase (Pearson coefficient = 0.54 and 0.76, respectively), and the activity of acid phosphatase was positively related to alkaline phosphatase (Pearson coefficient = 0.7).

## 4. Discussion

### 4.1. Effects of Soil Type

The soil requirements for optimal green bean growth are pH 5.0–6.5, >2 mg (100 g^−1^) P, >10 mg (100 g^−1^) K, 200–2000 mg kg^−1^ Ca, >120 mg kg^−1^ Mg and 10–50 mg kg^−1^ Na [51]. According to requirements, the pH of red and brown soils in this study was higher than optimal. Both soils in our study were in the optimal range for green bean growing based on the available P and K, with red soil being much higher in soil-available P and K (Table 1). However, red soil was more productive, as was demonstrated by the higher flower bud count. 

Negative correlation between root:shoot and soil-available P and K confirmed that green bean root growth has uptaken and, thus, lowered the supply of nutrients in the soil. The activities of acid and alkaline phosphatase were higher in brown soil with less phosphorous than in red soil with high phosphorous. Greater acid and alkaline phosphatase activity was reported on brown soil compared with red soil and confirmed the findings of Lemanowicz [52] that phosphatase activity increased when soil is low in available P since brown soil showed lower soil-available P content compared with red soil. That was similar to previously confirmed data of increased green bean growth and yield at high P nutrition [53] and high K and Mg nutrition [54]. Microorganisms can enhance P availability in soil by several mechanisms: the extension of root systems by hyfe of mycorrhizal fungi or by hormonal stimulation of root growth, alteration of sorption equilibria, induction of metabolic processes that release protons, organic anions and siderophores, phosphatases and cellulolytic enzymes that act on sparingly available inorganic and organic P substances [55].

Observed moderately negative correlation of active soil reaction (pH H_2_O) to respiration intensity and alkaline phosphatase activity suggested that microbial activity increased at lower pH. Bacterial growth decreased linearly, while fungal growth increased exponentially with soil pH from 8.3 to 4.5 [56]. In our study, soil respiration correlated with dehydrogenase and alkaline phosphatase activities, as was expected for indicators of microbial activity. Also, it was observed that soil microbial activity was affected the most by soil-available P and K, and pH.

The nettle application increased active soil acidity regardless of soil type and disinfection, while potential acidity (pH KCl) was lower in disinfected soils. The change in potential soil reaction could be attributed to the complex of different biochemical reactions on soil exchange sites that occurred after the disinfection with dazomet. However, Scharenbroch et al. [57] did not observe changes in pH, C, N, Mg, or Na in silty loam and clay loam soil following aerated compost tea application. 

### 4.2. Effects of Soil Disinfection

The higher above-ground growth and root dry weight of green beans after the disinfection with dazomet, regardless of soil type and irrigation, suggest that the changes in the microbial community were beneficial for green beans. That was opposed to the findings of Koron, Sonjak, and Regvar [58], where strawberry yield and growth was lower after dazomet application than after the application of two other techniques for soil disinfection (*Brassicaceae* green manure incorporation and heating) or control. An increase in lettuce growth after soil fumigation with dazomet reported by [59] Bonanomi et al. showed that the microbial community was negatively affected by dazomet application, while dazomet promoted the growth of the *Pseudomonas fluorescens* population. *Pseudomonas* can have a positive effect on plant growth by promoting rhizobacteria that promote plant nutrition, release stimulatory compounds, and act as a biocontrol agent against soil-borne pathogens. Other research reported increased growth of melon and increased soil N content as a result of dead microbial biomass [60]. Yan et al. [61] found that soil was richer in nutrients after soil fumigation with dazomet because of the mineralization of dead microbial biomass.

Observed higher root:shoot ratio in no-disinfected brown soil compared with no-disinfected red soil could be attributed to increased allocation of plant resources to roots in poor soil, while the decrease in root:shoot ratio after disinfection could be due to the presence of competition among the native microbial community of brown soil. Poorter and Nagel [62] found that different plants respond to a decrease in below-ground resources with increased allocation to roots. 

Root:shoot ratio was negatively related to specific root length (SRL, m g^−1^, Table 3), the parameter indicating plants with higher nutrients and water uptake efficiency [63]. Corneo et al. [64] found that plants with higher SRL have higher leaf ϭ13 C (indicating higher water use efficiency) and higher N leaf content. We observed greater SRL in red no-disinfected soil than in brown disinfected soil, which was probably due to higher nutrient content in red than in brown soil necessary for the plant to develop the denser root system. 

Greater content of soil-available P and K in red no-disinfected soil than in red disinfected soil, but not in brown disinfected and brown no-disinfected soil suggested that the effect of dazomet application to soil fertility differs based on previous soil status. In red soil, which was considered healthy soil, disinfection decreased soil-available P and K, while in brown soil, which was considered unhealthy, disinfection was not effective in the release of P and K. It is known that dazomet decreases the richness and diversity of soil bacteria and fungi [65]. 

### 4.3. Effects of Nettle Extract Application on Green Bean Growth

Based on the chemical composition of non-aerated nettle extract, each pot received 0.624 g N mineral, 0.06 g PO_4-_P, and 3.2 g K with 1 L of the extract or 62.4 kg ha^−1^ N, 5.7 kg ha^−1^ P and 320 kg ha^−1^ K during four weeks of the experiment. Nettle extract had a positive effect on the above-ground growth of green beans in our experiment, and the effect was observed on both soil types and disinfection treatments. The application of nettle extract increased studied vegetative and generative traits at the flowering stage. At the same time, the application of nettle extract showed different effects on root growth, depending on soil type and disinfection applied. Root growth was significantly increased at the harvest stage by nettle extract application on red soil. The findings of Godlewska et al. [18] were similar since among the 14 plant extracts studied, nettle leaf and root extracts were the most effective in stimulating shoot length, shoot fresh weight and shoot dry weight of white head cabbage seedlings. Likewise, Peterson and Jensen [10,37] found that aqueous nettle extract enhanced the fresh and dry weight of tomato shoots and barley compared with growing the same plants by adding mineral fertilizer. The same authors found that both root length and fresh weight of barley plants treated with nettle extract were two times larger than plants treated with mineral nutrient solution. They suggested that the cause for higher shoot mass was the plant hormone auxin, which is normally found in nettle-aqueous extract [6]. Some research also reported that nettle extract did not strongly affect vegetative growth, as was found by Rivera et al. [34]. In their study on the effect of aqueous nettle extract and onion extract (*Allium cepa* L.) applied through the soil in lettuce (*Lactuca sativa* L.) cultivation, onion extract enhanced the vegetative growth of lettuce compared to nettle extract.

The effect of plant extract on vegetable growth depends on the mode of application, whether it is foliar or through the soil. In green bean production at open field conditions, nettle extract applied through the soil did not affect vegetative parameters (mass of dry stem and leaves) compared with unfertilized soil [66]. On the other hand, foliar application of raw extracts from leaves and flowers of *Borago officinalis* L. on plants of *Lactuca sativa* ‘Longifolia’ enhanced yield and growth by 16% compared with control plants treated with water [15]. 

In this study, the nettle extract increased the yield of green beans by 48% compared with water irrigation. Dozet et al. [67] found that foliar application of aqueous *Urtica dioica* and *Pulmonaria officinalis* plant extracts increased the yield of soybean by only 9% compared to the control, while extract made solely of nettle increased yield even less, by 7%, compared with the control treatment. Similar to these findings, Pane et al. [7] found a significant increase in the yield of lettuce (24%) after the application of aerated compost tea made of artichokes and fennel and a higher yield of kohlrabi (32%) after foliar spraying.

### 4.4. Effects of Soil Type, Soil Disinfection and Nettle Extract Application Interaction on Green Bean Growth

Higher soil-available K was detected after nettle extract application on no-disinfected soil compared with nettle extract and water-irrigated disinfected soil. It suggested that in addition to the nutrients and microorganisms added with nettle application, native soil microorganisms were stimulated by nettle extract and thus activated the release of soil-available K in both soils. Lian et al. [68] found that K release from soil minerals was enhanced by the presence of thermophilic fungus. One of the most important findings of this study was that nettle extract application increased the alkaline phosphatase activity regardless of soil type and disinfection. 

Greater soil respiration at both soils irrigated with nettle extract compared with water-irrigated ones also suggested that nutrients and other components from the nettle extract improve soil microbial activity. Araújo et al. [69] found higher basal soil respiration in organic farming systems compared with conventional farming systems and native vegetation. The dehydrogenase activity in nettle extract-irrigated disinfected red soil was greater compared with water-irrigated disinfected red soil but similar in brown soil, which was probably attributed to microorganisms present in the nettle extract and the enhancement of the microbial community developed after the disinfection with dazomet on red soil. Dehydrogenase activity was found to correlate with the abundance of bacteria, fungi, and actinomycetes in soils where organic materials were applied [70,71]. Observed lower dehydrogenase activity in all water-treated soils and at all treatments on brown soil was probably attributed to the lack of substrate for dehydrogenase activity and the competition between microorganisms for the substrate. The lack of correlation between dehydrogenase activity and soil organic matter was expected since organic matter represented the easy and recalcitrant carbon compounds. This form of carbon is not available for microbial decomposition. Arriagada et al. [72] found that dehydrogenase activity was inhibited by microbial communities in soil inoculated with both mycorrhizae and saprobe fungi species because of higher competition between these microorganisms for available nutrients. Positive correlations between dehydrogenase activity and leaf area suggested that the enhanced microbial activity was beneficial for green bean vegetative growth.

Low root nodule number at all treatments, except on brown no-disinfected soil irrigated with water, could be attributed to the inhibition of nodulation by nitrogen compounds from nettle extract and the interactions between N-fixing bacteria and other soil microorganisms such as competition for limited nutrients, antagonism, predation, and parasitism [73]. Symbiotic N_2_ fixation was described as a facultative process that stops at high soil nitrogen content [74,75,76,77]. Contrary to this study, Kim et al. [78] found that aerated compost tea increased root nodulation of soybean. Although they applied another plant extract, the most important difference was the much lower application rate of nitrogen originating from 50 mL of plant extract per plant once a week. This research applied 1 L of plant extract per pot every two days. The results showed that in water-irrigated no-disinfected brown soil, the observed higher nodule count was not effective for stimulation of the above-ground growth, while nettle extract application on the same soil significantly increased green bean growth. According to Isoi and Yoshida [79], common beans (*Phaseolus vulgaris* L.) have low nitrogen fixation activity mostly due to the low weight of nodules and because of the strains of symbiotic bacteria *Rhizobium legumonosarum bv. phaseoli* found on common bean roots showed low nitrogen fixation activity.

## 5. Conclusions

The application of nettle extract had the combined effect of enriching the soil with nutrients and microorganisms and, thus, positively affected the vegetative and generative growth of green beans, although it negatively affected the root nodulation. Also, the application of nettle extract positively affected phosphorus nutrition by increasing alkaline phosphatase activity. The native microbial community was positively affected by the application of nettle extract and stimulated the release of soil-available K, as well as soil respiration. Furthermore, the release of soil-available P was also positively affected by nettle extract application, probably due to increased alkaline phosphatase activity. The application of soil disinfection enhanced green bean vegetative and generative growth, probably as a result of the death of pathogenic microorganisms and the following change in the microbial community. The combination of soil disinfection and nettle water application stimulated dehydrogenase activity in red soil but not in brown soil. It happened possibly due to the lower amount of available nutrients and easily available organic carbon, which should be present in order to allow the nettle extract to affect the growth of the microbial community positively.

In this study, green beans showed a tendency to increase root:shoot ratio on poorer soils (such as the studied brown soil) as an important mechanism implemented by crops when coping with nutrient deficits. Overall, this research revealed the complexity of the mechanisms by which organic fertilizers act. The optimization of the nettle extract dose under greenhouse and field conditions would be the next step toward the implementation of nettle extract as an organic fertilizer and soil improver.

## Figures and Tables

**Table 1:** Chemical analysis of soil.

Soil Type	pH (H_2_O)	pH (KCl)	N (%)	P (mg 100 g^−1^)	K (mg 100 ^g−1^)	Humus (%)
Red soil	7.99 ± 0.04 ^a^	7.24 ± 0.01 ^a^	0.17 ± 0.01 ^a^	31.05 ± 0.61 ^a^	32.25 ± 1.80 ^a^	2.75 ± 0.08 ^a^
Brown soil	7.85 ± 0.03 ^b^	7.20 ± 0.01 ^b^	0.12 ± 0.01 ^b^	5.46 ± 0.84 ^b^	13.55 ± 0.65 ^b^	2.02 ± 0.11 ^b^

Different letters mark statistically different treatments at a 0.05 level of significance.

**Table 2:** Chemical composition of undiluted nettle extract prepared in this study compared with standard Hoagland’s nutrient solution [42].

Parameter	Unit	Nettle Extract	Nettle Extract (Peterson and Jensen, 1985)	Hoagland’s Nutrient Solution
pH		6.53	5.50–5.85	6.00
EC	mS/cm	5.42	4.25–5.02	1.80
NH_4_-N	mM	8.90	13.60–18.40	2.00
NO_2,_ NO_3_-N	mM	0.02	0.00–0.20	14.50
P	mM	0.37	1.30–4.00	2.00
K	mM	16.20	9.50–44.40	6.50
Ca	mM	15.20	10.90–18.30	4.00
Mg	mM	2.40	2.40–3.30	2.00
S	mM	1.60	2.30–2.40	2.00
Fe	µM	3.23	48.30–118.10	45.00
B	µM	n.a. *	71.20–99.00	4.60
Mn	µM	0.62	21.10–22.00	0.50
Zn	µM	3.80	11.50–26.70	0.20
Cu	µM	1.08	6.00–8.00	0.20
Mo	µM	n.a.	0.90–6.00	0.70

* n.a.—not analyzed.

**Table 3:** Soil pH, organic matter (OM), and soil-available phosphorus (P_2_O_5_) and potassium (K_2_O) at soil type, disinfection, and irrigation treatments; mean ± standard error, the significance of tree-way ANOVA.

Factor	Level	pH (H_2_O)	pH (KCl)	OM (mg g^−1^)	P_2_O_5_ (mg 100 g^−1^)	K_2_O (mg 100 g^−1^)
Soil (S)	Brown	7.78 ± 0.04	7.20 ± 0.02	10.60 ± 0.57	3.50 ± 0.81	32.10 ± 4.05
	Red	7.84 ± 0.03	7.17 ± 0.02	10.60 ± 0.22	16.30 ± 0.85	49.00 ± 5.73
Disinfection (D)	Yes	7.83 ± 0.04	7.16 ± 0.02	10.40 ± 0.40	9.10 ± 2.00	38.90 ± 4.60
	No	7.79 ± 0.03	7.21 ± 0.01	10.80 ± 0.45	10.70 ± 3.00	42.30 ± 6.90
Irrigation (I)	Nettle	7.74 ± 0.02	7.20 ± 0.02	11.10 ± 0.20	11.00 ± 2.20	52.90 ± 4.35
	Water	7.88 ± 0.03	7.17 ± 0.02	10.10 ± 0. 51	8.80 ± 2.80	28.30 ± 2.72
Significance						
S		0.065	0.027	1.000	0.001	0.001
D		0.195	0.002	0.439	0.001	0.079
I		0.001	0.027	0.057	0.001	0.001
SxD		0.002	0.168	0.443	0.001	0.006
SxI		0.520	0.622	0.347	0.001	0.060
DxI		0.073	0.338	0.138	0.770	0.039
SxDxI		0.164	0.001	0.137	0.707	0.442

**Table 4:** Green beans shoot biomass, root biomass, and shoot/root ratio at soil type, disinfection, and irrigation treatments; mean ± standard error, the significance of tree-way ANOVA performed after alignment and ranking of variables.

Factor	Level	Shoot Biomass (g)	Root Biomass (mg)	Root:Shoot
Soil (S)	Brown	1.03 ± 0.12	330.00 ± 19.00	0.34 ± 0.03
	Red	1.17 ± 0.08	290.00 ± 30.00	0.26 ± 0.03
Disinfection (D)	Yes	1.22 ± 0.09	350.00 ± 17.00	0.30 ± 0.02
	No	0.97 ± 0.10	270.00 ± 23.00	0.30 ± 0.11
Irrigation (I)	Nettle	1.29 ± 0.09	310.00 ± 27.00	0.26 ± 0.03
	Water	0.95 ± 0.07	310.00 ± 25.00	0.34 ± 0.028
Significance				
S		0.140	0.440	0.032
D		0.009	0.014	0.073
I		0.004	1.000	0.032
SxD		0.061	0.770	0.032
IxS		0.300	1.000	0.350
IxD		0.230	0.880	0.760
IxSxD		0.590	0.490	0.093

**Table 5:** Green beans height, leaf area, flower buds, shoot dry weight, root dry weight, ratio shoot/root specific root length and root nodule count at soil type, disinfection, and irrigation treatments; mean ± standard error, the significance of tree-way ANOVA performed after alignment and ranking of variables.

Factor	Level	Leaf Area (cm^2^)	Flower Bud Count	Root Nodule Count
Soil (S)	Brown	210.00 ± 30.00	4.86 ± 0.51	90.60 ± 47.00
	Red	248.00 ± 32.00	5.80 ± 0.46	84.00 ± 20.00
Disinfection (D)	Yes	273.00 ± 29.00	5.94 ± 0.46	128.00 ± 42.00
	No	181.00 ± 23.00	4.71 ± 0.46	47.00 ± 20.00
Irrigation (I)	Nettle	292.00 ± 28.00	6.39 ± 0.37	32.60 ± 8.00
	Water	176.00 ± 16.00	4.46 ± 0.35	142.00 ± 41.00
Significance				
S		0.240	0.006	0.820
D		0.004	0.004	0.001
I		0.001	0.001	0.001
SxD		0.710	0.160	0.002
IxS		0.540	1.000	0.310
IxD		0.710	0.260	0.007
IxSxD		0.940	0.230	0.014

**Table 6:** Green beans total yield, shoot and root dry weight, and pod length and diameter at soil type, disinfection, and irrigation treatments; mean ± standard error, the significance of tree-way ANOVA performed at the end of the experiment.

Factor	Level	Pods Length (cm)	Pods Diameter (mm)	Total Yield (g)	Shoot Dry Weight(g)	Root Dry Weight (g)
Soil (S)	Brown	8.68 ± 0.18	6.79 ± 0.15 ^b^	60.85 ± 13.46 ^b^	5.53 ± 0.30	2.03 ± 0.06 ^b^
	Red	9.06 ± 0.16	7.41 ± 0.12 ^a^	90.70 ± 8.26 ^a^	6.21 ± 0.39	3.12 ± 0.17 ^a^
Disinfection (D)	Yes	9.27 ± 0.12 ^a^	7.26 ± 0.12	104.06 ± 10.46 ^a^	6.46 ± 0.28 ^a^	2.64 ± 0.18
	No	8.36 ± 0.21 ^b^	6.90 ± 0.17	47.49 ± 7.93 ^b^	5.29 ± 0.37 ^b^	2.51 ± 0.16
Irrigation (I)	Nettle	9.29 ± 0.14 ^a^	7.38 ± 0.14 ^a^	90.51 ± 12.16 ^a^	6.79 ± 0.30 ^a^	3.77 ± 0.22 ^a^
	Water	8.39 ± 0.18 ^b^	6.79 ± 0.13 ^b^	61.04 ± 10.11 ^b^	4.96 ± 0.29 ^b^	2.37 ± 0.10 ^b^
Significance						
S		0.07654	0.00013	0.013703	0.063119	0.000000
D		0.000037	0.0651	0.000037	0.002095	0.412978
I		0.00003	0.00177	0.014797	0.000007	0.019376
SxD		0.23331	0.00011	0.174397	0.208528	0.876521
IxS		0.76466	0.40371	0.830372	0.809223	0.003867
IxD		0.02593	0.31899	0.629620	0.011883	0.852882
IxSxD		0.1022	0.98859	0.364287	0.905730	0.718366

Different letters mark statistically different treatments at a 0.05 level of significance.

**Table 7:** The significant interactions between soil type, disinfection, and irrigation treatment on pod diameter, root dry weight, shoot dry weight, and pod length at the end of the experiment.

	Pods Diameter (mm)		Root Dry Weight (g)	
	Disinfection		Irrigation	
Soil	Yes	No	Nettle	Water
Brown	7.27 ± 0.16 ^a^	6.22 ± 0.22 ^b^	1.97 ± 0.10 ^c^	2.08 ± 0.08 ^c^
Red	7.26 ± 0.17 ^a^	7.62 ± 0.16 ^a^	3.57 ± 0.26 ^a^	2.66 ± 0.13 ^b^
	**Pods length (cm)**		**Shoot dry weight (g)**	
	**Irrigation**		**Irrigation**	
Disinfection	Nettle	Water	Nettle	Water
Yes	9.48 ± 0.19 ^a^	9.03 ± 0.15 ^a^	6.91 ± 0.43 ^a^	6.01 ± 0.34 ^a^
No	9.05 ± 0.22 ^a^	7.62 ± 0.31 ^b^	6.67 ± 0.43 ^a^	3.90 ± 0.19 ^b^

Different letters mark statistically different treatments at a 0.05 level of significance.

**Table 8:** Activity of dehydrogenases and acid and alkaline phosphatase at soil type, disinfection, and irrigation treatments; mean ± standard error, the significance of tree-way ANOVA performed after alignment and ranking of variables.

Factor	Levels	Dehydrogenases (nmol g^−1^ h^−1^ TPF)	Acid Phosphatase (µmol g^−1^ h^−1^ pNP)	Alkaline Phosphatase (µmol g^−1^ h^−1^ pNP)	Soil Respiration (mg CO_2_ kg^−1^)
Soil (S)	Brown	25.60 ± 1.10	1.15 ± 0.07	3.62 ± 3.20	889 ± 38
	Red	23.20 ± 3.50	0.93 ± 0.06	2.29 ± 1.80	723 ± 80
Disinfection (D)	Yes	26.60 ± 3.10	1.07 ± 0.05	3.04 ± 2.40	785 ± 65
	No	22.10 ± 1.60	1.01 ± 0.10	2.87 ± 2.10	828 ± 74
Irrigation (I)	Nettle	27.70 ± 2.60	1.11 ± 0.10	3.34 ± 2.70	945 ± 35
	Water	21.10 ± 2.00	0.97 ± 0.05	2.57 ± 1.90	667 ± 55
Significance					
S		0.078	0.037	0.0011	0.00415
D		0.010	0.71	0.31	0.334
I		0.0023	0.20	0.0011	0.00016
SxD		0.0065	0.20	0.17	0.479
SxI		0.0011	0.71	0.54	0.0284
DxI		0.12	0.44	0.71	0.249
SxDxI		0.0011	0.65	0.17	0.834

**Table 9:** Correlations matrix between soil parameters: electrical conductivity (EC), active acidity (pH H_2_O), potential acidity (pH KCl), available P (P_2_O_5_), available K (K_2_O), organic matter (OM), respiration (CO_2_), dehydrogenase activity (DHA), acid phosphatase (AcP) and alkaline phosphatase (AlP). Values represent the Pearson correlation coefficient; marked coefficients indicate significant correlations at *p* < 0.05 in the regression analysis.

	EC	DHA	AcP	AlP	%C	Ph H_2_O	pH KCl	P_2_O_5_	K_2_O	CO_2_	Plant Height	Stem Diameter	Leaf Area	Flower no.	Shoot Dry Weight	Root Nodule Count	Spec Root Length	Root:shoot	Root Dry Weight
EC	1																		
DHA	0.32	1																	
AcP	0.45	0.48	1																
AlP	0.75	0.55	0.75	1															
OM	0.52	0.05	0.01	0.15	1														
pH H_2_O	−0.85	−0.38	−0.44	−0.58	−0.6	1													
pH KCl	0.56	−0.28	0.03	0.26	0.44	−0.42	1												
P_2_O_5_	−0.32	−0.26	−0.51	−0.74	0.07	0.21	−0.13	1											
K_2_O	0.35	0.19	−0.13	−0.09	0.25	−0.3	0.22	0.68	1										
CO_2_	0.76	0.54	0.47	0.76	0.16	−0.56	0.42	−0.3	0.43	1									
Plant height	0.22	0.34	−0.14	−0.08	0.21	−0.26	−0.06	0.58	0.84	0.31	1								
Stem diameter	0.28	0.03	0.09	0.07	−0.03	−0.19	0.17	−0.02	0.18	0.06	0.26	1							
Leaf area	0.21	0.54	0.02	0.11	0.24	−0.31	−0.16	0.24	0.57	0.31	0.83	0.02	1						
Flower no.	0.18	0.48	0.02	0.07	0.12	−0.26	−0.27	0.41	0.66	0.32	0.92	0.07	0.86	1					
Shoot dw	0.13	0.28	−0.11	0.01	0.21	−0.16	−0.1	0.37	0.54	0.11	0.84	0.18	0.84	0.84	1				
Root nodule count	−0.57	−0.21	−0.25	−0.23	−0.58	0.79	−0.51	−0.09	−0.39	−0.37	−0.17	−0.09	−0.17	−0.07	−0.02	1			
Spec root length	−0.13	−0.37	−0.53	−0.47	0.22	0.1	0.23	0.51	0.27	−0.26	0.2	0.29	−0.14	−0.09	0.16	−0.11	1		
Root:shoot	−0.05	−0.04	0.27	0.2	−0.29	0.12	−0.08	−0.58	−0.57	0.02	−0.68	−0.18	−0.5	−0.53	−0.78	0.26	−0.66	1	
Root dw	0.03	0.27	0.27	0.28	−0.3	0.08	−0.4	−0.32	−0.16	0.12	0.05	−0.1	0.28	0.32	0.1	0.46	−0.83	0.52	1

## References

[1] Kuepper G. (2010). A Brief Overview of the History and Philosophy of Organic Agriculture.

[2] Godlewska K., Biesiada A., Michalak I., Pacyga P. (2019). The Effect of Plant-Derived Biostimulants on White Head Cabbage Seedlings Grown under Controlled Conditions. Sustainability.

[3] Soil Health. NRCS Soils. https://www.nrcs.usda.gov/wps/portal/nrcs/main/soils/health/.

[4] Palčić I., Jagatić Korenika A.M., Jakobović S., Pasković I., Major N., Ban D., Ban S.G., Karoglan M., Petek M., Herak Ćustić M. (2020). Soil type affects grape juice free amino acids profile during ripening of cv. Malvasia Istriana (*Vitis vinifera* L.). N. Z. J. Crop Hortic. Sci..

[5] Yim B., Smalla K., Winkelmann T. (2013). Evaluation of apple replant problems based on different soil disinfection treatments—links to soil microbial community structure?. Plant Soil.

[6] Peterson R., Jensen P. (1985). Effects of nettle water on growth and mineral nutrition of plants. I. composition and properties of nettle water. Biol. Agric. Hortic..

[7] Pane C., Palese A.M., Celano G., Zaccardelli M. (2014). Effects of compost tea treatments on productivity of lettuce and kohlrabi systems under organic cropping management. Ital. J. Agron..

[8] Mohd Din A.R.J., Cheng K.K., Sarmidi M.R. (2017). Assessment of compost extract on yield and phytochemical contents of Pak Choi (*Brassica rapa* cv. *chinensis*) grown under different fertilizer strategies. Commun. Soil Sci. Plant Anal..

[9] Ji R., Dong G., Shi W., Min J. (2017). Effects of liquid organic fertilizers on plant growth and rhizosphere soil characteristics of chrysanthemum. Sustainability.

[10] Peterson R., Jensen P. (1986). Effects of nettle (*Urtica dioica*) water on growth and mineral nutrition of plants: II. Pot-culture and water-culture experiments. Biol. Agric. Hortic..

[11] Kandeler E., Tscherko D., Spiegel H. (1999). Long-term monitoring of microbial biomass, N mineralisation and enzyme activities of a Chernozem under different tillage management. Biol. Fertil. Soils.

[12] Chang E.H., Chung R.S., Tsai Y.H. (2007). Effect of different application rates of organic fertilizer on soil enzyme activity and microbial population. Soil Sci. Plant Nutr..

[13] Adeyeye A.S., Togun A.O., Olaniyan A.B., Akanbi W.B. (2017). Effect of fertilizer and rhizobium inoculation on growth and yield of soyabean variety (*Glycine max* L. Merrill). Adv. Crop Sci. Technol..

[14] Canfora L., Malusà E., Salvati L., Renzi G., Petrarulo M., Benedetti A. (2015). Short-term impact of two liquid organic fertilizers on *Solanum lycopersicum* L. *rhizosphere* Eubacteria and Archaea diversity. Appl. Soil Ecol..

[15] Bulgari R., Morgutti S., Cocetta G., Negrini N., Farris S., Calcante A., Negrini N., Farris S., Calcante A., Spinardi A. (2017). Evaluation of borage extracts as potential biostimulant using a phenomic, agronomic, physiological, and biochemical approach. Front. Plant Sci..

[16] Calvo P., Nelson L., Kloepper J.W. (2014). Agricultural uses of plant biostimulants. Plant Soil.

[17] Du Jardin P. (2015). Plant biostimulants: Definition, concept, main categories and regulation. Sci. Hortic..

[18] Godlewska K., Biesiada A., Michalak I., Pacyga P. (2020). The Effect of Botanical Extracts Obtained through Ultrasound-Assisted Extraction on White Head Cabbage (*Brassica Oleracea* L. var. *Capitata* L.) Seedlings Grown under Controlled Conditions. Sustainability.

[19] Ertani A., Pizzeghello D., Francioso O., Tinti A., Nardi S. (2016). Biological activity of vegetal extracts containing phenols on plant metabolism. Molecules.

[20] Merwad A.R.M. (2018). Using *Moringa oleifera* extract as biostimulant enhancing the growth, yield and nutrients accumulation of pea plants. J. Plant Nutr..

[21] Gülçin I., Küfrevioǧlu Ö.İ., Oktay M., Büyükokuroǧlu M.E. (2004). Antioxidant, antimicrobial, antiulcer and analgesic activities of nettle (*Urtica dioica* L.). J. Ethnopharmacol..

[22] Yıldız L., Başkan K.S., Tütem E., Apak R. (2008). Combined HPLC-CUPRAC (cupric ion reducing antioxidant capacity) assay of parsley, celery leaves, and nettle. Talanta.

[23] Ahangarpour A., Mohammadian M., Dianat M. (2012). Antidiabetic effect of hydroalcholic *Urtica dioica* leaf extract in male rats with fructose-induced insulin resistance. Iran J. Med. Sci..

[24] Upton R. (2013). Stinging nettles leaf (*Urtica dioica* L.): Extraordinary vegetable medicine. J. Herb. Med..

[25] Bisht S., Bhandari S., Bisht N.S. (2012). *Urtica dioica* (L): An undervalued, economically important plant. Agric. Sci. Res. J..

[26] Bacci L., Baronti S., Predieri S., di Virgilio N. (2009). Fiber yield and quality of fiber nettle (*Urtica dioica* L.) cultivated in Italy. Ind. Crops Prod..

[27] Di Virgilio N., Papazoglou E.G., Jankauskiene Z., Di Lonardo S., Praczyk M., Wielgusz K. (2015). The potential of stinging nettle (*Urtica dioica* L.) as a crop with multiple uses. Ind. Crops Prod..

[28] Amini S., Azizi M., Joharchi M.R., Shafei M.N., Moradinezhad F., Fujii Y. (2014). Determination of allelopathic potential in some medicinal and wild plant species of Iran by dish pack method. Theor. Exp. Plant Physiol..

[29] Maričić B., Radman S., Romić M., Perković J., Major N., Urlić B., Palčić I., Ban D., Zorić Z., Ban S.G. (2021). Stinging Nettle (*Urtica dioica* L.) as an Aqueous Plant-Based Extract Fertilizer in Green Bean (*Phaseolus vulgaris* L.) Sustainable Agriculture. Sustainability.

[30] Bozsik A. (1996). Studies on aphicidal efficiency of different stinging nettle extracts. Anz. Schädlingskd. Pfl. Umwelt..

[31] Kaberia D.K. (2007). Participatory Action Research and Testing the Effectiveness of Stinging Nettle as a Biopesticide in Kenya. Ph.D. Thesis.

[32] Hadizadeh I., Peivastegan B., Kolahi M. (2009). Antifungal activity of nettle (*Urtica dioica* L.), colocynth (*Citrullus colocynthis* L. Schrad), oleander (*Nerium oleander* L.) and konar (*Ziziphus spina-christi* L.) extracts on plants pathogenic fungi. Pak. J. Biol. Sci..

[33] Nygaard Sørensen J., Thorup-Kristensen K. (2011). Plant-based fertilizers for organic vegetable production. J. Plant. Nutr. Soil Sci..

[34] Rivera M.C., Wright E.R., Salice S., Fabrizio M.C. (2012). Effect of plant preparations on lettuce yield. Acta Hortic..

[35] Otles S., Yalcin B. (2012). Phenolic compounds analysis of root, stalk, and leaves of nettle. Sci. World J..

[36] Zeipiņa S., Alsiņa I., Lepse L. (2014). Stinging nettle–the source of biologically active compounds as sustainable daily diet supplement. Res. Rural Dev..

[37] Peterson R., Jensen P. (1988). Uptake and transport of nitrogen, phosphorus and potassium in tomato supplied with nettle water and nutrient solution. Plant Soil.

[38] Ganesan K., Xu B. (2017). Polyphenol-rich dry common beans (*Phaseolus vulgaris* L.) and their health benefits. Int. J. Mol. Sci..

[39] Chávez-Mendoza C., Sánchez E. (2017). Bioactive compounds from Mexican varieties of the common bean (*Phaseolus vulgaris*): Implications for health. Molecules.

[40] Lešić R., Borošić J., Buturac I., Herak Ćustić M., Poljak M., Romić D. (2016). Povrćarstvo.

[41] Bogunović M., Vidaček Ž., Racz Z., Husnjak S., Špoljar A., Sraka M. (1998). FAO/Unesco. Pedološka Karta 1:1.000.000.

[42] Hoagland D.R., Arnon D.I. (1950). The water-culture method for growing plants without soil. Circ. Calif. Agric. Exp. Stn..

[43] Al-Busaidi A., Cookson P., Yamamoto T. (2005). Methods of pH determination in calcareous soils: Use of electrolytes and suspension effect. Aust. J. Soil Res..

[44] FAO (2019). Standard Operating Procedure for Soil Organic Carbon. Walkley-Black Method. Titration and Colorimetric Method, GLOSOLAN-SOP-02, Food and Agriculture Organization of the United Nations. http://www.fao.org/3/ca7471en/CA7471EN.pdf.

[45] Zebec V., Rastija D., Lončarić Z., Bensa A., Popović B., Ivezić V. (2017). Comparison of chemical extraction methods for determination of soil potassium in different soil types. Eurasian Soil Sci..

[46] Kargas G., Chatzigiakoumis I., Kollias A., Spiliotis D., Kerkides P. (2018). An Investigation of the relationship between the electrical conductivity of the soil saturated paste extract ECe with the respective values of the mass soil/water ratios 1: 1 and 1: 5 (EC1: 1 and EC1: 5). Proceedings.

[47] Loeppert R.H., Suarez D.L., Sparks D.L., Page A.L., Helmke P.A., Loeppert R.H., Soltanpour P.N., Tabatabai M.A., Johnston C.T., Sumner M.E. (1996). Carbonate and gypsum. Methods of Soil Analysis: Part 3 Chemical Methods.

[48] Tabatabai M.A., Bottomley P.J., Angle J.S., Weaver R.W. (1994). Soil enzymes. Methods of Soil Analysis: Part 2 Microbiological and Biochemical Properties.

[49] Wolinska A., Stępniewska Z., Szymańska E. (2013). Dehydrogenase activity of soil microorganisms and the total DNA level in soil of different use. J. Agric. Sci. Technol. B.

[50] Rubio V.E., Detto M. (2017). Spatiotemporal variability of soil respiration in a seasonal tropical forest. Ecol. Evol..

[51] Green Bean Production Guideline. https://www.starkeayres.com/uploads/files/Bean-Production-Guideline-2019.pdf.

[52] Lemanowicz J. (2011). Phosphatases activity and plant available phosphorus in soil under winter wheat (*Triticum aestivum* L.) fertilized minerally. Pol. J. Agron..

[53] Moghaddam F.R., Aminpanah H. (2015). Green bean (*Phaseolus vulgaris* L.) growth and yield as affected by chemical phosphorus fertilizer and phosphate bio-fertilizer. Idesia.

[54] Huda A.I., El-Behairy U.A., El-Desuki M., Bakry M.O., Abou-Hadid A.F. (2010). Response of green bean to fertilization with potassium and magnesium. Res. J. Agric. Biol. Sci..

[55] Richardson A.E., Simpson R.J. (2011). Soil microorganisms mediating phosphorus availability update on microbial phosphorus. Plant Physiol..

[56] Rousk J., Brookes P.C., Baath E. (2009). Contrasting soil pH effects on fungal and bacterial growth suggest functional redundancy in carbon mineralization. Appl. Environ. Microbiol..

[57] Scharenbroch B.C., Johnston D.P. (2011). A microcosm study of the common night crawler earthworm (*Lumbricus terrestris*) and physical, chemical and biological properties of a designed urban soil. Urban Ecosyst..

[58] Koron D., Sonjak S., Regvar M. (2014). Effects of non-chemical soil fumigant treatments on root colonisation with arbuscular mycorrhizal fungi and strawberry fruit production. Crop Prot..

[59] Bonanomi G., Chiurazzi M., Caporaso S., Del Sorbo G., Moschetti G., Felice S. (2008). Soil solarization with biodegradable materials and its impact on soil microbial communities. Soil Biol. Biochem..

[60] Yamamoto T., Ultra Jr. V. (2008). U.; Tanaka, S.; Sakurai, K.; Iwasaki, K. Effects of methyl bromide fumigation, chloropicrin fumigation and steam sterilization on soil nitrogen dynamics and microbial properties in a pot culture experiment. Soil Sci. Plant Nutr..

[61] Yan D., Wang Q., Mao L., Ma T., Li Y., Ouyang C., Guo M., Cao A. (2015). Interaction between nitrification, denitrification and nitrous oxide production in fumigated soils. Atmos. Environ..

[62] Poorter H., Nagel O. (2000). The role of biomass allocation in the growth response of plants to different levels of light, CO_2_, nutrients and water: A quantitative review. Funct. Plant Biol..

[63] Pérez-Harguindeguy N., Díaz S., Garnier E., Lavorel S., Poorter H., Jaureguiberry P., Bret-Harte M.S., Cornwell W.K., Craine J.M., Gurvich D.E. (2013). New handbook for standardised measurement of plant functional traits worldwide. Aust. Bot..

[64] Corneo P.E., Keitel C., Kertesz M.A., Dijkstra F.A. (2017). Variation in specific root length among 23 wheat genotypes affects leaf δ13C and yield. Agric. Ecosyst. Environ..

[65] Wang Q., Ma Y., Yang H., Chang Z. (2014). Effect of biofumigation and chemical fumigation on soil microbial community structure and control of pepper Phytophthora blight. World J. Microbiol. Biotechnol..

[66] Pasković I., Radić T., Perinčić B., Užila Z., Palčić I., Ban D., Romić M., Žnidarčić D., Ban S.G. The effect of aqueous nettle extract on soil fertility and dwarf French bean vegetative growth. Proceedings of the 52. Croatian and 12. International Symposium on Agriculture.

[67] Dozet G., Ðukić V., Balešević-Tubić S., Ðurić N., Miladinov Z., Vasin J., Jakšić S. (2017). Uticaj primene vodenih ekstrakata na prinos u organskoj proizvodnji soje. Zbornik Radova 1, XXII Savetovanje o Biotehnologiji sa Međunarodnim Učešćem.

[68] Lian B., Wang B., Pan M., Liu C., Teng H.H. (2008). Microbial release of potassium from K-bearing minerals by thermophilic fungus Aspergillus fumigatus. Geochim. Cosmochim. Acta.

[69] Araújo A.S., Leite L.F., Santos V.B., Carneiro R.F. (2009). Soil microbial activity in conventional and organic agricultural systems. Sustainability.

[70] Janušauskaite D., Kadžienė G., Auškalnienė O. (2013). The effect of tillage system on soil microbiota in relation to soil structure. Pol. J. Environ. Stud..

[71] Bhatt B., Chandra R., Ram S., Pareek N. (2016). Long-term effects of fertilization and manuring on productivity and soil biological properties under rice (*Oryza sativa*)—Wheat (*Triticum aestivum*) sequence in Mollisols. Arch. Agron. Soil Sci..

[72] Arriagada C., Manquel D., Cornejo P., Soto J., Sampedro I., Ocampo J. (2012). Effects of the co-inoculation with saprobe and mycorrhizal fungi on Vaccinium corymbosum growth and some soil enzymatic activities. J. Soil Sci. Plant Nutr..

[73] Kapoor K.K., Dudeja S.S., Mishra P.C., Behera N., Senapati B.K., Guru B.C. (1995). Ecology of legume root nodule bacteria. Advances in Ecology and Environmental Sciences.

[74] Adak M.S., Kibritci M. (2016). Effect of nitrogen and phosphorus levels on nodulation and yield components in faba bean (*Vicia faba* L.). Legume Res..

[75] Liese R., Schulze J., Cabeza R.A. (2017). Nitrate application or P deficiency induce a decline in Medicago truncatula N2-fixation by similar changes in the nodule transcriptome. Sci. Rep..

[76] Pérez-Fernández M.A., Calvo-Magro E., Rodríguez-Sánchez J., Valentine A. (2017). Differential growth costs and nitrogen fixation in *Cytisus multiflorus* (L′ Hér.) Sweet and *Cytisus scoparius* (L.) Link are mediated by sources of inorganic N. Plant Biol..

[77] Regus J.U., Wendlandt C.E., Bantay R.M., Gano-Cohen K.A., Gleason N.J., Hollowell A.C., O’Neill M.R., Sachs J.L. (2017). Nitrogen deposition decreases the benefits of symbiosis in a native legume. Plant Soil.

[78] Kim M.J., Shim C.K., Kim Y.K., Hong S.J., Park J.H., Han E.J., Kim J.H., Kim S.C. (2015). Effect of aerated compost tea on the growth promotion of lettuce, soybean, and sweet corn in organic cultivation. Plant Pathol. J..

[79] Isoi T., Yoshida S. (1991). Low nitrogen fixation of common bean (*Phaseolus vulgaris* L.). Soil Sci. Plant Nutr..

